# In *Mycoplasma hominis *the OppA-mediated cytoadhesion depends on its ATPase activity

**DOI:** 10.1186/1471-2180-11-185

**Published:** 2011-08-19

**Authors:** Miriam Hopfe, Theresa Dahlmanns, Birgit Henrich

**Affiliations:** 1 Institute of Medical Microbiology and Hospital Hygiene, Heinrich-Heine-University Duesseldorf, Moorenstrasse 5, 40225 Duesseldorf, Germany

## Abstract

**Background:**

In *Mycoplasma homini*s, a facultative human pathogen of the human genital tract, OppA, the substrate-binding domain of the oligopeptide permease, is a multifunctional protein involved in nutrition uptake, cytoadhesion and hydrolysis of extracellular ATP.

**Results:**

To map the function-related protein regions the ATPase activity and adhesive behavior of OppA mutants were analyzed. Mutations of the Walker BA motifs resulted in an inhibition of up to 8% of the OppA ATPase activity, whereas deletion of the N-terminal CS1 or the CS2 region, structural motifs that are conserved in bacterial OppA proteins, reduced ATPase activity to 60% and deletion of CS3, the third conserved region adjacent to the Walker B motif led to a reduction to 42% ATPase activity.

Interestingly, adhesion of the OppA mutants to immobilized HeLa cells demonstrated that two distal regions are mainly involved in adherence of OppA: the CS1 region, deletion of which led to 35% of the cytoadhesion, and the Walker BA with the adjacent upstream region CS3, deletion of which led to 25% of the cytoadhesion. The influence of the ATPase activity on the adherence of *M. hominis *to HeLa cells was confirmed by the use of ATPase inhibitors which reduced mycoplasmal cytoadhesion to 50%.

**Conclusions:**

These findings suggest that the OppA-mediated cytoadherence of *Mycoplasma homini*s depends on both, the topology of the neighbouring CS1 and ATPase domain regions and the functionality of the ecto-ATPase activity in addition.

## Background

Adherence to host tissues is an essential and complex stage of bacterial colonization preceding the establishment of a bacterial infection. Therefore analysis of surface exposed proteins is a very important step in providing more information about the mechanisms of adhesion, colonization and invasion of host tissues as well as of the ability of the organism to evade the host immune system.

A large number of Gram-negative and Gram-positive bacteria use fimbriae and pili for bacterial attachment [1]. In mycoplasmas, which belong to the class of mollicutes characterized by the lack of a cell wall, fimbrial structures are missing. Hence, mycoplasmal membrane proteins exposed to the external environment mediate direct binding of the bacteria to host cells. Surface exposed structures like lipids [2-4], membrane proteins [5,6] and lipoproteins [6-10] must be considered as potential cytoadherence factors.

*Mycoplasma hominis *is a facultative pathogen of the human urogenital tract. *In silico *analysis of the *M. hominis *genome led to an annotation of 537 proteins. The minimal set of 220 proteins postulated to be essential for survival of this mycoplasma species [11] includes the cytoadhesive lipoproteins P50, also known as variable adherence associated antigen [12], P60, a domain of a membrane complex [6], and OppA, the substrate-binding domain of the oligopeptide permease [13]. Over the past years OppA of *M. hominis *has been characterized as a multifunctional protein, the functions of which include: 1. the substrate-binding domain of the oligopeptide permease [13]; 2. it acts as an immunogenic cytoadhesin, whose binding to HeLa cells is inhibited in the presence of the monoclonal antibody BG11 [6]; and 3. it represents the main Mg^2+^-dependent ecto-ATPase which is a unique feature of *M. hominis *in contrast to OppA proteins of other mollicutes [14]. Using *in vitro *infection assays the pathophysiological role of OppA has become obvious as its ecto-ATPase activity was shown to induce ATP release from HeLa cells and their subsequent death [15].

Based on the sequence characteristics of this ATPase domain, OppA belongs to the class of P-loop NTPases whose nucleotide binding fold is composed of a conserved Walker A motif (a so called P-loop) and a less conserved Walker B motif. These are both generally found in the cytoplasmic ATP-hydrolyzing domains of ABC-transporters as motors for transport [16]. The ATPase domain of OppA is remarkable in that the order of Walker A and B on the polypeptide chain is inverted to Walker B and A. To date this orientation has only been found in the ATPase binding fold of myosin in rabbits and nematodes [17]. With regard to other P-loop NTPases, OppA of *M. hominis *is the only one localized on the surface [18]. In other pro- and eukaryotic ecto-NTPases, the P-loop structure is missing and in these instances nucleotide binding is mediated by a different structure characterized by conserved ACR-regions first described in apyrase [19]. Despite structural differences in the catalytic domains, common features with OppA include their extracellular localization, the ability to hydrolyze ATP with a high turnover (Km 200 - 400 μM), and their dependence on divalent cations.

To date mammalian ecto-ATPases have been shown to be involved in several cell functions: 1. protection from the cytolytic effect of extra-cellular ATP [20,21], 2. regulation of ecto-kinases by modulation of ATP-content as a substrate [22], 3. involvement in signal transduction [22-24], and 4. cellular adhesion [25,26]. In parasites like *Trypanosoma cruzi *it has been shown that an enhanced expression in ecto-ATPase activity leads to a concomitant increase in adhesion to macrophages whereas its inhibition abrogates adhesion and internalisation by these host cells [25,26].

In the present work the relationship of the two OppA-functions, ATPase activity and cytoadherence, was analyzed. We show that the cytoadhesion of *M. hominis *is dependent on the ecto-ATPase activity of OppA and that this could be assigned to distinct regions of the protein.

## Results

### Generation of recombinant OppA mutants modified in putative functional sites

**Figure 1:**
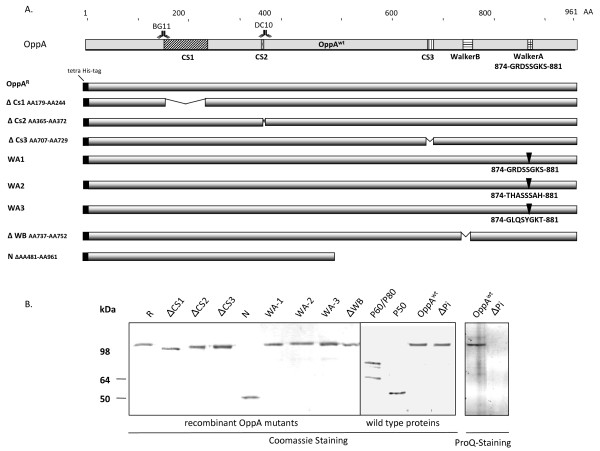
**OppA variants**. A. Schematical overview of proposed functional OppA regions and secondary structure predictions of the OppA variants. The binding sites of mAb BG11 and mAb DC10 are depicted with antibody icons. CS1, a conserved region of bacterial OppA proteins, is shown in diagonal strips, and conserved regions of mycoplasmal OppA proteins are depicted by dotted areas (CS2) and vertical strips (CS3). The ATP-binding site consists of the C-terminal localized Walker A (grid) and Walker B (horizontal strips) motifs. The deletion mutants were sign with gaps between the OppA bulks. Modified regions of the Walker A mutants were described below the OppA bulks. B. SDS-PAGE of the recombinant OppA mutants and wild type proteins P50, P60/P80, OppA^wt ^and the dephosphorylated OppA^ΔPi ^variant. The purified proteins were separated on a 9.5% SDS gel followed by Coomassie staining and the wild type OppA variants in addition by ProQ- staining demonstrating phosphorylations. SeeBlue Plus 2 Pre-Stained Standard from Invitrogen was used as molecular weight marker.

To dissect which regions of the OppA polypeptide chain might determine adhesion and its ATPase activity, recombinant OppA mutants were constructed (Figure 1A).

In the search for conserved sequence motifs in OppA proteins of different species, three regions with high homologies were detected: the region of AA179 - AA244, which is conserved in bacterial OppA proteins, thus named CS1 (consensus sequence 1), and regions CS2 (AA365 - AA372) and CS3 (AA701 - AA729), which are conserved in mycoplasmal OppA proteins. To determine the functions of these regions, OppA mutants, OppA^ΔCS1^, OppA^ΔCS2 ^and OppA^ΔCS3 ^were constructed (Figure 1A).

With regard to the ATPase activity of OppA we analyzed five mutants. In 2004 two OppA mutants, OppA^K875R ^(here named OppA^WA1^) and OppA^ΔP-loop ^(OppA^WA2^) had already been characterized. They were modified to different extent within the Walker A region (AA869 - AA876) leading to a decreased ATPase activity to 15% (OppA^WA1^) and 6% (OppA^WA2^) in relation to the wild type [14]. As computer analysis revealed a putative Walker A motif (AA411 - AA418) in the OppA protein of *M. pulmonis *(MYPU_6070), we constructed a third Walker A mutant (OppA^WA3^) by replacing the original Walker A region of *M. hominis *with the putative Walker A sequence. Interestingly this putative Walker A motif of *M. pulmonis *OppA is located within the CS2 region. In the fourth OppA mutant, OppA^ΔWB ^the less conserved Walker B motif plus a downstream region of several hydrophobic amino acids was deleted (AA737 - AA752). In the OppA^N ^mutant the C-terminal half of OppA (AA481- AA 961) was deleted thus missing the CS3, Walker B and Walker A motif. All OppA mutants were expressed in *E. coli *with an N-terminal histidine-tag instead of the 28 AA signal peptide; including the cysteine residue where signal peptidase II cleavage and lipid modification would normally take place in *M. hominis*. After purification the quality of the OppA mutants and wild type membrane proteins used in the following analyses was documented by SDS- PAGE. Dephosphorylation of OppA was demonstrated by ProQ staining (Figure 1B).

### ATPase activity of the OppA mutants

**Figure 2:**
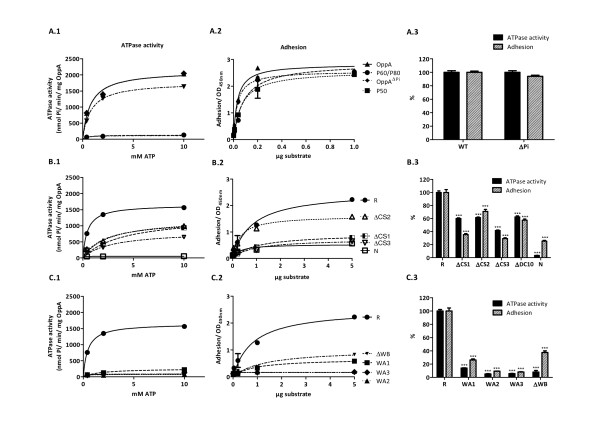
**ATPase activity and adhesion of *M. hominis *membrane proteins P50, P60/P80 and OppA variants**. ATPase activities of purified proteins (0.5 μg/well) were measured in the ammonium molybdate assay as a function of ATP concentration [A.1-C.1] Protein adhesion to HeLa cells was measured in cell-ELISA [A.2-C.2]. A comparison of the relative ATPase activity (black bars) and adhesion (striped bars) with regard to wild type OppA is shown in [A.3-C.3]. Data represent means of three independent experiments with triplicate samples in each experiment. Statistical analysis was performed by unpaired t-test and statistically significant results designated by *. *P < 0.05, **P < 0.01, and ***P < 0.001. The ATPase activity or adhesion of the OppA mutants were compared with those of the recombinant OppA (R).

First the kinetics of ATP hydrolysis of the different OppA mutants was analyzed by measuring the release of free phosphate (Figure 2A.1-C.1). The addition of MgATP to the OppA mutants led to an increase in ATPase activity in a dose-dependent and saturable manner. The data of ATP hydrolysis were fed into Michaelis-Menten equation. In nonlinear regression analysis the Michaelis constant, K_m _for the recombinant OppA^R ^was 0.46 ± 0.04 mM ATP, whereas K_m _for the wild type OppA^WT ^was 0.18 ± 0.04 mM. As the Michaelis constant behaves reciprocally to the enzyme affinity this exhibits a higher affinity of OppA^WT ^for ATP than OppA^R^. This may be due to a partial misfolding of the recombinant variant. However, the maximum reaction rate (V_max _1543 ± 32.54 nmol/min/mg) was similar for both proteins.

As shown in Figure 2A.1 dephosphorylation of OppA had no influence on its ATPase activity (K_m _0.39 ± 0.04 mM ATP) whereas mutations within either the Walker A or Walker B motifs led to a dramatic decrease in ATP-hydrolysis. As previously shown in 2004 [14] a single point mutation in the Walker A motif (K875R) led to a decreased ATP-hydrolysis by OppA^WA1 ^to 15% whereas ATP-binding still occurred. Mutation of the whole Walker A motif in OppA^WA2 ^resulted in the complete inhibition of both ATP-binding and hydrolysis. Exchanging the Walker A motif of *M. hominis *with the putative Walker A sequence of *M. pulmonis *in OppA^WA3 ^also led to inhibition of the ATP-hydrolysis indicating that the Walker A motif of *M. pulmonis *in this context is non-functional. As expected both the OppA-mutant lacking the Walker B motif (OppA^ΔWB^) as well as the OppA^N ^-mutant with a complete deletion of the C- terminal half of OppA, including the ATP-binding domain, did not show any ATPase activity (Figure 2C.1).

Next we examined the contribution of the other conserved regions on the catalytic function of OppA. Deletion of the CS2 region (AA365-372) led to an increased K_m _in the OppA^ΔCS2^mutant (2.56 ± 0.43 mM ATP) (Figure 2B.1). With regard to the OppA^ΔCs1 ^and OppA^ΔCs3 ^mutants the lowest affinity for ATP was observed for the OppA^ΔCs3 ^mutant (Km 2.86 ± 0.43), demonstrating a significant participation of the CS3 region in conformation of the neighbored Walker BA region.

These data provide evidence that in addition to the Walker A and B motif the conserved regions CS3, CS1, and CS2 affect ATPase activity (in descending order) and suggest that these regions are involved in stabilizing the catalytic ATPase domain of OppA.

### ATPase domain of OppA mediates cytoadherence

Participation of the well characterized membrane proteins P50, P60/P80 and OppA (P100) in cytoadherence of *Mycoplasma hominis *had previously been demonstrated by comparing the binding capacity of the purified proteins to immobilized HeLa cells with cytoadherence of *M. hominis *cells [6]. The cell ELISA was used to scrutinize the OppA binding more closely in which the membrane proteins P50, P60/P80 and OppA served as positive controls. As shown in Figure 2A.2, the membrane proteins attached to HeLa cells in a dose-dependent manner. Nonlinear regression and one-site binding analyses were performed to estimate the apparent dissociation constants for P50 (0.07 ± 0.01 μg), P60/P80 (0.08 ± 0.02 μg), OppA (0.03 ± 0.01 μg) and dephosphorylated OppA^ΔPi^-variant (0.03 ± 0.03). Deletion of the CS2 region (AA365 - AA372) reduced adhesion of the OppA^R ^to 70% (Figure 2B.2) whereas deletion of either the CS1 region (in OppA^ΔCS1^) or the C-terminal half of OppA (in OppA^N^) led to a decrease in adherence to 35% and 25%, respectively, suggesting a high impact of the Walker BA region on cytoadhesion. This was affirmed by analysis of the other Walker BA mutants of OppA (Figure 2C.2). As mutations of the Walker A motif in OppA^WA2 ^and OppA^WA3 ^inhibited binding of OppA to 9% and 8%, respectively, the P-loop structure was demonstrated as an essential part for OppA-adhesion (Figure 2C.2). These findings are summarized in Figure 2[A.3-C.3] depicting the ATPase activity and the adhesive regions of the respective OppA mutant in relation to OppA and suggest that the presence and interaction of the N-terminal localized CS1 region with the catalytic site of the ATPase domain (composed of the CS3 region and the Walker BA regions) take part in OppA's attachment of HeLa cells.

**Figure 3:**
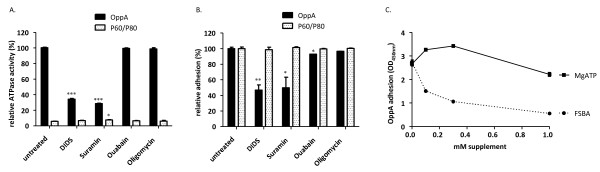
**Adherence of OppA to HeLa cells in the presence of ATPase inhibitors**. OppA (black bars) or P60/P80 as a control (white bars), (0.5 μg OppA/well and 0.3 μg P60/well) were preincubated with 200μM DIDS, suramin, ouabain or oligomycin for 20 min before analyzing in adhesion assay. ATPase activity (A) and adhesion efficiency (B) were measured and depicted in relation to the untreated OppA. OppA (0.5 μg protein) was preincubated with FSBA or MgATP for 20 min and then added to HeLa cells (C). Adherence of OppA to HeLa cells in dependence on supplement concentration was determined as described in Material and Methods. Data represent means of three independent experiments with triplicate samples in each experiment. Statistical analysis was performed by unpaired t-test and statistically significant results designated by *. *P < 0.05, **P < 0.01, and ***P < 0.001.

### OppA adherence depends mainly on ATP-hydrolysis

To ascertain whether binding of OppA depends not only on a conserved ATPase domain architecture but in fact on ATP hydrolysis, OppA binding to HeLa cells was characterized in the presence of DIDS (4,4'-diisothiocyano-2,2'-stilbene-disulfonic acid), an inhibitor of ecto- ATPases; suramin, an inhibitor of Ca^2+^-ATPases; oligomycin, an inhibitor of F_1_-ATPases; and ouabain, an inhibitor of Na^+^/K^+^-dependent ATPases.

OppA was neither able to hydrolyze ATP (Figure 3A) nor to attach to HeLa cells in the presence of DIDS and suramin (Figure 3B). This is in accordance with the findings that even cytoadherence of *M. hominis *to living HeLa cells was abolished by DIDS and suramin [14]. As expected oligomycin, an inhibitor of F1-ATPases, and ouabain, an inhibitor of ATPases dependant on monovalent cations, had neither an effect on ATPase activity of OppA nor on its adhesion to HeLa cells. Predictably, adherence of the *M. hominis *P60/P80 membrane protein complex lacking an ATPase activity remained unaffected by these inhibitors (Figure 3A and 3B).

To test the hypothesis that attachment of OppA is an energy-consuming step provided by ATPase hydrolysis we added FSBA (5'-p-fluorosulfonylbenzoyladenosine), a non-hydrolyzing adenosine, to the adhesion assay. ATP hydrolysis as well as adhesion of OppA to HeLa cells were competitively reduced in a dose-dependent manner to approximately 30% showing that ATP hydrolysis is essential for adhesion of OppA (Figure 3C). Moreover, OppA adherence to vital HeLa-cells decreased in the presence of ATP in concentrations of 0.1-0.3 mM whereas concentrations up to 1 mM MgATP inhibited adherence of OppA to HeLa.

## Discussion

With the observation that in the cell-wall less, facultative human-pathogen *Mycoplasma hominis*, OppA is a multifunctional lipoprotein involved in cytoadhesion, nutrition uptake and ecto-ATPase-mediated damage of the host cell, we started to map the cytoadhesive regions in relation to the ATPase domain on the polypeptide chain. Utilizing recombinant OppA mutants we observed that ecto-ATPase activity and adherence to HeLa cells are inter-dependent functions of OppA. Both functions are mainly influenced by the Walker A motif, supported by the Walker B motif and the upstream CS3 region for maximal ATPase activity, and maintained by the CS3 and CS1 regions in terms of adherence. These findings suggest an interaction or juxtaposition of these regions in the three-dimensional structure of the molecule, important for ATPase activity and attachment to the host, and clearly demonstrate that the OppA-mediated cytoadherence depends on autologous ATP-hydrolysis.

Bacterial OppA proteins usually function solely as substrate-binding domains of oligopeptide permeases. Oligopeptide importers (OppABCDF) belong to the class of ATP-binding-cassette- (ABC-) transporters with two pore-forming domains (OppBC) and two cytoplasmic ATPases (OppDF) [27]. The cytoplasmic ATPase domains function as motors for substrate transport across the membrane using ATP as fuel. It seems to be a freak of nature that in *M. hominis*, OppA has gained an additional ATPase activity which raises the question as to its function. To date ecto-ATPase activity of OppA is unique to *M. hominis *among substrate-binding proteins of ABC-transporters of all three kingdoms. Thus it seems illogical that the ecto-ATPase is required for optimized peptide import. The findings of this study clearly demonstrate that the OppA ecto-ATPase is essential for maximal cytoadhesion of *M. hominis*. In studying bacterial adhesion to polymer surfaces Stollenwerk and coworkers found that under conditions of starvation - by incubation in nutrient-poor buffer - the ATP content of adherent bacteria decreased after 24 h to 96 h whereas that of planktonic bacteria remained stable for up to 20 days [28]. This suggests that cytoadhesion is an energy-consuming process. Similar to our results presented here an ecto-ATPase-dependent cytoadherence has already been suggested for *Trypanosoma cruzi *whose ATPase activity was strongly inhibited by using DIDS or suramin attended by a reduced adhesion to mouse resident macrophages [25]. Early work of Bredt and coworkers in the 1980's demonstrated that cytoadhesion of the cell wall-less mollicutes is modulated by ATP. By monitoring the ATP content in the supernatant attachment of *M. pneumoniae *to glass surfaces was shown to depend on an intact energy metabolism [29]. In using a glucose-inhibitor, the ATP content declined and attachment was abrogated. In using an ATPase inhibitor, ATP content accumulates leading to a decreased cytoadherence. Bredt and coworkers hypothesized that the first step of colonization is energy dependent either to energize the membrane thus increasing some binding sites on the surface, or to modulate the contractile cytoskeleton [29]. The free energy of ATP hydrolysis by P-loop NTPases is typically utilized to introduce conformational changes in other molecules [30].

As adhesion of mycoplasmal cytoadhesins does not depend on ATP-hydrolysis at all, as demonstrated in this study for the P60/P80 membrane complex of *M. hominis*, ATPase dependent adhesion of OppA is predicted to play a special role in *M. hominis*. In 2008 OppA was shown to mediate apoptosis, to induce ATP-efflux and a concomitant ATP-depletion of the *M. hominis*-colonized host cell [15]. This is in accordance to the recent findings that the cytoadherence of *M. pneumoniae *induces an ATP-efflux from the colonized host [31]. ATP- efflux was considered as a stress-associated danger signal as it stimulates P2X_7_-receptors of the host leading to the expression of pro-inflammatory cytokines. It is well known that extracellular ATP signals through P2 receptors to modulate the immune and inflammatory response in a variety of host cells, including immune and non-immune cells, sometimes leading to apoptosis or necrosis of the cells [32]. Zhang and Lo demonstrated in 2007 that the principally invasive pathogens *M. fermentans *and *M. penetrans *prevent apoptosis and stimulate host cell growth of infected cells whereas the predominantly surface-colonizing species *M. hominis *and *M. salivarium *promote apoptosis [33]. Inhibition of P2X_7_-signaling appears to be more important for intracellular pathogens as shown by the treatment of *M. tuberculosis *infected macrophages with ATP, which results in killing of both the intracellular mycobacteria and the host, whereas conditions such as complement-mediated cytolysis, Fas ligation, and CD69 activation induced only lysis of the macrophages while preserving the bacterial vitality [34-36].

With regard to the findings that *M. hominis*, a well known colonizer of epithelial surfaces, has also been found in the intracellular compartment in cultured HeLa cells [37], *Trichomonas vaginalis *[38] and human spermatozoa [39], OppA-mediated cytoadhesion of *M. hominis *may play a key role in invasion. In case of infection the extracellular ATP-level is increased. Thus, an OppA-mediated decrease of this danger signal, thus preventing P2X_7 _- mediated signaling, with concomitant cytoadhesion are proposed mechanisms for mycoplasma survival to circumvent host immune defense mechanisms and facilitate invasion.

## Conclusions

The present study demonstrates that the enzymatic function of OppA as main ecto-ATPase of *M. hominis *is essential for adhesion and suggests that the unique feature of this mycoplasma has an impact on patho-physiological important processes in host-pathogen interactions.

## Methods

### HeLa cell culture

The human cervical carcinoma cell line HeLa S3 (ATCC CCL2.2) was obtained from the American Type Culture Collection (Rockville, MD, USA) and cultivated in Dulbecco's Modified Eagle Medium (Invitrogen GmbH, Darmstadt, Germany) with 10% horse serum (PAA laboratories GmbH, Pasching, Austria.)

### Mycoplasma culture conditions and purification of proteins

The *M. hominis *strains FBG was grown in PPLO broth base medium containing 1% (w/w) arginine as described previously [40]. Stocks were prepared from a mid-logarithmic-phase broth culture and stored in 1 ml portions at -70°C. For the purification of distinct proteins, cells of 1 L mid-logarithmic-phase broth culture were sedimented (10.000 × g, 20 min, 4°C) and the sediment washed twice with PBS and resuspended in 10 ml PBS. After protein concentration was estimated by Bradford analysis [41] and adjusted to 1 mg protein/ml PBS, membrane proteins were solubilised by 0.5% (w/v) N-dodecylmaltoside (Roche, Grenzach- Wyhlen, Germany). After 1 h incubation on a rotation wheel followed by centrifugation (15.000 × g, 20 min, RT), the supernatant was incubated with sepharose-coupled antibodies DC10, BG2 or CG4 and the respective proteins OppA, P50 and P60/P80 were isolated as previously described [6].

### Dephoshorylation of wild type OppA

2 μg OppA were incubated with 5 units shrimp alkaline phosphatase in 50 μl [10 mM Tris/HCl, pH 7.5, 50 mM NaCl] for 45 min at 37°C followed by acetone precipitation by adding 3 volumes of ice-cold acetone to the sample and incubating at -20°C overnight. The precipitated proteins were sedimented by centrifugation (13,000 × g, 20 min, 4°C) and residual acetone removed by air drying. The dephosphorylation status was verified by SDS- PAGE [42] and subsequent ProQ staining as described by the manufacturer's instructions (Invitrogen GmbH, Darmstadt, Germany).

### DNA manipulations

All routine DNA manipulation techniques, including plasmid preparation, restriction, ligation and transformation of *E. coli *were performed as described by [43] or according to the manufacturers' instructions.

The pXB-plasmids encoding protein C-tagged proteins OppA^R^, OppA^WA1 ^and OppA^WA2 ^[14] were used as targets for the construction of pQE30-plasmids expressing His-tagged OppA mutants. To facilitate cloning of the PCR products, restriction sites were flanked to the primer sequences without changing the encoded amino acid sequence (Table 1). For each mutant two primer pairs were used to generate two PCR-fragments, which were subsequently fused by SOE (splicing by overlap extension)-PCR [44] and cloned into the pQE30 vector.

**Table 1:** Primer used for the generation of OppA mutants

oppA clone	deletion/mutation (AA)	name	primer sequence (5'-3')	annealing (°C)
ΔCS1	Δ176-243	OppA start	5'-GTGGCGGCCGCGCCTGCAGTTTTTTAG-3'	60°C
		CS1 down	5'-TCTTGATTCAACGTTCTTGTCACCT-3'	60°C
		CS1 up	5'- AAGAACGTTGAATCAAGAGAACTAGATGAAGC-3'	62°C
		OppA end	5'-GGTCCATGGTGGGTACCAAAATAGACCCGGCATATGTAAAA-3'	62°C

ΔCS2	Δ365-372	OppA start	5'-GTGGCGGCCGCGCCTGCAGTTTTTTAG-3'	50°C
		CS2 down	5'-TGAGACGTCTGTAAGCTATCTTTATCCATTGAA-3'	50°C
		CS2 up	5'-AAAGATAGCTTACAATACGCTAAATCTACATTG-3'	62°C
		OppA end	5'-GGTCCATGGTGGGTACCAAAATAGACCCGGCATATGTAAAA-3'	62°C

ΔDC10	Δ366-381	OppA start	5'-GTGGCGGCCGCGCCTGCAGTTTTTTAG-3'	58°C
		DC10 down	5'-CTGACCAATTTTGTATTGTAAGCTATCT-3'	58°C
		DC10 up	5'-TACAAAATTGGTCAGAAAGGTATAGAAAAC-3'	58°C
		OppA end	5'-GGTCCATGGTGGGTACCAAAATAGACCCGGCATATGTAAAA-3'	58°C

ΔCS3	Δ647-675	OppA start	5'-GTGGCGGCCGCGCCTGCAGTTTTTTAG-3'	61°C
		CS3 down	5'-GTACAGCTGTGGAGCATTTAAATATCT-3'	61°C
		CS3 up	5'-GCTCCACAGCTGTACGATCCAAACTTCAA-3'	60°C
		OppA end	5'-GGTCCATGGTGGGTACCAAAATAGACCCGGCATATGTAAAA-3	60°C

ΔWB	Δ712-727	OppA start	5'-GTGGCGGCCGCGCCTGCAGTTTTTTAG-3'	50°C
		DC10 down	5'-ATATGCGTTGAAGTTTGGAT-3'	50°C
		DC10 up	5'-TATAACGGTGTTGCTAGCACATAC-3'	58°C
		OppA end	5'-GGGTCCATGGTGGGTACCAAAATAGACCCGGCATATGTAAAA-3'	58°C

WA3	874GKDSSGKS-GLQSYGKT881	OppA start	5'-GTGGCGGCCGCGCCTGCAGTTTTTTAG-3'	60°C
		DC10 down	5'-TACAGATCTGTTGGTTCTATAGTTTTTCCATAACTCTGCAATCCAAAATC-3'	60°C
		DC10 up	5'-CAACAGATCTGTATCAGTGGTCTGCAAT-3'	60°C
		OppA end	5'-GGGTCCATGGTGGGTACCAAAATAGACCCGGCATATGTAAAA-3'	60°C

### *Escherichia coli *strains

*E. coli *strain DH5α (Invitrogen, Darmstadt, Germany) was used for cloning whereas strain *E. coli *strain BL21-Lys (Novagen-Merck, Darmstadt, Germany) was used for expression of recombinant peptides.

### Expression and purification of recombinant proteins

The His-tagged OppA variants were expressed in *E. coli *and purified to homogeneity by metal chelating chromatography using Ni(II)-NTA-resin. 200 ml LB-broth medium (Gibco BRL, Gaithersburg, Md) containing ampicillin (100 μg/ml) was inoculated with 20 ml of overnight culture of the respective *E. coli *BL 21-Lys clone for 1 h at 37°C with vigorous shaking until an OD_600 nm _of 0.6 to 0.9 was reached. Protein expression was induced by isopropylthio-β-D- galactoside (0.2 mM). After 3 h of shaking at 37°C the cells were harvested by centrifugation (15,000 × g, 20 min, 4°C) and frozen at -20°C. After thawing on ice the cells were resuspended in 17 ml buffer A [20 mM Tris/HCl pH 8.0, 500 mM KCl, 10 mM imidazole, 10 mM β-mercaptoethanol, 10% [v/v] glycerol, 5% [w/v] N-lauroylsarcosine, 1 tablet protease inhibitor (Roche, Grenzach-Wyhlen, Germany)] and incubated for 2 h on a rotating wheel followed by one burst of sonication on ice (5 min at 95 W). The lysate was centrifuged (15,000 × g, 20 min, 4°C) and the supernatant transferred to 0.5 ml 50% slurry of Ni-NTA- sepharose (Qiagen, Hilden, Germany) and incubated for 4 h at RT on a rotating wheel. The sepharose was loaded into a 1 cm diameter column and washed with 20 ml washing buffer [20 mM Tris/HCl pH 8.0, 500 mM KCl, 10 mM imidazole, 10 mM β-mercaptoethanol, 10% [v/v] glycerol, 0.5% [w/v] N-lauroylsarcosine]. The bound proteins were eluted from the Ni-NTA resin by using wash buffer supplemented with 150 mM imidazole. 10 fractions of 0.5 ml were collected and 20 μl of each fraction analyzed on 9.5% polyacrylamide gels [42].

### Adhesion assays

The adhesion assays with wild type proteins of *M. hominis *(OppA, P50, the P60/P80 membrane complex) and the recombinant OppA mutants were performed as a cell ELISA according to the description of Henrich *et al*., 1993 [6] with the following modifications: HeLa cells (1 × 10^5 ^cells/well) were immobilized with 1.25% (v/v) glutaraldehyde to lysine- coated 96-well micro-plates (Greiner Bio-one GmbH, Frickenhausen, Germany) as described formerly [45] and incubated in DMEM^FCS ^[DMEM 10% (v/v) fetal bovine serum] (Lonza Cologne GmbH, Cologne, Germany) for 30 min at 37°C. The proteins were serial diluted 1:5 in DMEM^FCS^, using a starting concentration of 1 μg protein/well for the wild type proteins and 5 μg protein/well for the OppA mutants, and incubated with the immobilized HeLa cells for 2 h at 37°C.

To analyze the influence of ATPase inhibitors the OppA protein or *M. hominis *cells were preincubated for 20 min with DIDS, Suramin, Ouabain, Oligomycin, FSBA or MgATP (Sigma) in concentrations as written in the figure legends before incubating with the HeLa cells.

After removal of unbound protein by washing twice with DMEM^FCS ^adherent wild type proteins were detected by protein-specific antibodies as described formerly [6]. For the detection of His-tagged OppA mutants monoclonal tetra-His antibody (Qiagen, Hilden, Germany) was used.

### ATP hydrolysis assay

The ATPase assay was performed as described by Hopfe and Henrich, 2004 [14], using an ammonium molybdate solution to quantify the emerging free phosphates.

### Sequence analysis

Analyses of DNA and protein sequences and design of oligonucleotides were facilitated by the Lasergene software package of DNA star Inc. (Madison, Wis.). Homology searches were done by Blast analysis http://blast.ncbi.nlm.nih.gov. *In silico *secondary structure analyses of the OppA variants were performed by the SOPMA Secondary Prediction Method (Pôle BioInformatique Lyonnaise network proteon sequence analysis; http://npsa-pbil.ibcp.fr/cgi-bin/npsa_automat.pl?page=npsa_sopma.html)

### Statistical analysis

All experiments were performed in triplicate, with similar results obtained by at least three independent tests. K_m _and V_max _were calculated with a computerized nonlinear regression analysis (Graph Pad Prism, version 5.01; Graph Pad Software Inc. Sang Diego, Calif.).

## Authors' contributions

MH carried out all experimental part and analysed the data. TD performed PCR analyses and sequencing of the OppA^ΔBG11 ^gene sequence. BH participated in the design and co-ordination of the study. MH and BH drafted the manuscript. All authors read and approved the final manuscript.

## Funding

This work was supported by a grant from the research commission of the medical faculty of the Heinrich-Heine University Duesseldorf, Germany.
